# Dr. Joseph T. Cox

**Published:** 1918-12

**Authors:** 


**Dr. Joseph T. Cox,** Chicago Homoeopathic 1888, Health Officer of Penn Yan, died of influenza Oct. 24, having been exposed in his official duties.

